# Evaluation of the “Los Filabres” Protocol on Behavioral and Psychological Symptoms of Dementia and Psychotropic Drug Use in Nursing Home Residents

**DOI:** 10.3390/healthcare14131934

**Published:** 2026-07-01

**Authors:** Isaac García Carricondo, Ana Rocío García Carricondo, Raúl Romero Del Rey, Raquel Alarcón-Rodríguez

**Affiliations:** 1 Department of Nursing, Los Filabres Nursing Home, 04550 Gérgal, Almería, Spain; isaacgarciacarricondo@gmail.com; 2 Oral and Maxillofacial Surgery Clinical Management Unit, Torrecárdenas University Hospital, 04009 Almería, Spain; argcarricondo@hotmail.es; 3 Department of Nursing, Physiotherapy and Medicine, Faculty of Health Sciences, University of Almería, 04120 Almería, Spain; ralarcon@ual.es; 4 Research Group CTS-1127 “Epidemiology and Public Health”, University of Almería, 04120 Almería, Spain; 5 Health Research Center (CEINSA), University of Almería, 04120 Almería, Spain

**Keywords:** behavioral and psychological symptoms of dementia, dementia care, person-centered care, non-pharmacological interventions, psychotropic drug use, nursing homes, unmet needs

## Abstract

**Highlights:**

**What are the main findings?**

- Significant longitudinal reductions in BPSD were observed during implementation of the “Los Filabres” protocol in nursing home residents with dementia.
- Longitudinal reductions were observed in psychotropic drug use, particularly antipsychotics and anxiolytics.

**What are the implications of the main findings?**

- Structured, person-centered non-pharmacological interventions may support improvements in BPSD and dementia care.
- Addressing unmet needs may support more individualized care and reduce reliance on psychotropic medications in long-term care settings.

**Abstract:**

**Background/Objectives:** Behavioral and psychological symptoms of dementia (BPSD) are highly prevalent among nursing home residents and represent a major clinical and care-related challenge. These symptoms are frequently associated with increased psychotropic drug use despite limited efficacy and important safety concerns. This study aimed to evaluate longitudinal changes in BPSD and psychotropic drug use following implementation of the “Los Filabres” protocol, a structured person-centered non-pharmacological intervention, in nursing home residents with dementia. **Methods:** A single-arm longitudinal pre–post observational study was conducted in five nursing homes in Andalusia, Spain, including 204 residents with dementia or cognitive impairment. After staff training, the intervention was implemented over 12 months. Outcomes were assessed at baseline (T0), 6 months (T1) and 12 months (T2) using the Neuropsychiatric Inventory (NPI), including symptom frequency, severity, clinical relevance, and caregiver-related distress. Psychotropic drug use was analyzed according to the Anatomical Therapeutic Chemical classification system. Statistical analyses included Friedman and Cochran’s *Q* tests, with effect sizes estimated using Cohen’s *d* and *h*. The observational nature of the study implies that observed changes may be subject to limitations such as Hawthorne effects. **Results:** Significant reductions were observed across all global NPI dimensions over time (*p* < 0.001), including symptom frequency, severity, clinical relevance, and caregiver-related distress. The proportion of participants with at least one clinically relevant symptom decreased from 93.1% at baseline to 35.8% at 12 months (*p* < 0.001). Significant longitudinal reductions were also observed in psychotropic drug use, with the mean number of psychotropic drugs per participant decreasing from 2.38 to 1.57 (*p* < 0.001). Reductions were observed in anxiolytic and antipsychotic use, as well as in as-needed prescriptions. **Conclusions:** The “Los Filabres” protocol was associated with significant longitudinal reductions in BPSD and psychotropic drug use in nursing home residents with dementia. These findings suggest that structured person-centered non-pharmacological interventions based on the identification of unmet needs may help support dementia care and more individualized pharmacological management in long-term care settings. These findings should be interpreted cautiously due to the observational pre–post design and absence of a control group.

## 1. Introduction

Behavioral and psychological symptoms of dementia (BPSD) represent one of the main clinical and care-related challenges in dementia management. It is estimated that more than 90% of individuals with dementia will develop at least one BPSD throughout the disease course [1], with prevalence rates reaching up to 97% in Alzheimer’s disease [2]. These symptoms include agitation, apathy, depression, anxiety, aggression, delusions, and hallucinations, among others, and are associated with increased morbidity, mortality, early institutionalization, caregiver burden, and significantly higher healthcare and social costs [3,4,5]. Their presence negatively impacts the quality of life of affected individuals and complicates both formal and informal caregiving, representing a key determinant of stress, fatigue, and emotional burden among care staff.

The etiology of BPSD is multifactorial, resulting from the interaction of neurobiological, psychological, social, and environmental factors. These symptoms should be understood as expressions of distress or unmet needs; therefore, their management should focus on identifying and addressing these needs, rather than attempting to suppress symptoms through restrictive or pharmacological interventions [1,4,6].

Despite international recommendations advocating for personalized, non-pharmacological interventions as the first-line treatment for BPSD [1,7,8,9,10], real-world practice in long-term care settings continues to show a persistent trend toward overmedication. More than 25% of institutionalized individuals with dementia receive long-term psychotropic treatment, with particularly widespread use of antipsychotics and benzodiazepines [1]. This prescribing pattern persists despite limited efficacy and well-documented risks of serious adverse effects, including cognitive decline, falls, cerebrovascular events, and increased mortality [11]. In contrast, accumulated evidence indicates that individualized non-pharmacological approaches can reduce the frequency and severity of BPSD, improve quality of life, and often decrease the use of psychotropic medication [5,8,9]. Approaches based on individualized behavioral analysis have demonstrated effectiveness by identifying personal and environmental determinants, including unmet needs that may trigger or maintain BPSD [4,6], leading to better outcomes in symptom reduction, improved caregiver–patient relationships, and reduced emotional burden of care [5,8,9,10]. BPSD are commonly assessed using structured instruments such as the Neuropsychiatric Inventory (NPI) [12], a widely used tool that evaluates the frequency and severity of the most prevalent neuropsychiatric symptoms associated with dementia.

Multicomponent programs such as DICE (Describe, Investigate, Create, and Evaluate), WHELD (Well-being and Health for People with Dementia), and STA OP! have demonstrated efficacy by integrating principles of Person-Centered Care and structured behavioral assessments [3,13,14]. The DICE [3] model focuses on an iterative case management process, while WHELD [13] emphasizes staff training and person-centered activities, and STA OP! [14] proposes a multidisciplinary stepped-care approach for advanced dementia. The “Los Filabres” protocol is distinguished by its integration of Virginia Henderson’s 14 basic human needs model as its central analytical framework, with structured clinical language so that the cause of the symptom can be precisely identified. Furthermore, unlike the aforementioned interventions, the protocol incorporates a clinical decision-making algorithm that prioritizes an initial differential assessment phase, distinguishing BPSD from acute confusional states or situational reactions, which provides an operational guide specifically adapted to the complexity of daily clinical practice in residential settings. Under these premises, the present study aimed to analyze the association between the implementation of the “Los Filabres” protocol and changes in behavioral and psychological symptoms in institutionalized individuals with dementia, considering not only the number of symptoms but also their frequency, severity, clinical relevance, and caregiver-related distress, as well as the evolution of psychotropic drug use.

## 2. Materials and Methods

### 2.1. Study Design

A longitudinal observational study was conducted to evaluate the changes associated with the intervention on neuropsychiatric symptoms, as well as on psychotropic drug use. This study was reported in accordance with the Strengthening the Reporting of Observational Studies in Epidemiology (STROBE) guidelines [15]. In addition, the intervention was described following the Template for Intervention Description and Replication (TIDieR) checklist [16].

### 2.2. Setting

The study was carried out in five nursing homes located in the Autonomous Community of Andalusia (Spain). These are long-term care facilities providing accommodation, daily living support, and comprehensive care for dependent older adults, operating 24 h a day, 7 days a week [17]. All participating facilities maintained their routine care standards; no additional structured non-pharmacological interventions targeting BPSD or major organizational changes were implemented during the 12-month study period.

### 2.3. Participants

The study population consisted of 204 residents from the participating nursing homes, recruited between January 2024 and December 2025. Inclusion criteria were: (a) diagnosis of dementia or presence of cognitive impairment (specified or unspecified); (b) permanent residence in the facility for at least three months prior to baseline data collection; and (c) ability to provide informed consent or, where applicable, availability of a legal representative. Exclusion criteria were: (a) a score of 3 or below on the Global Deterioration Scale (GDS) [18]. The GDS was used to stage the severity of primary degenerative dementia, with a score of 4 or higher required for inclusion to ensure the sample represented moderate to severe stages of the disease; (b) hospital admission within the previous three months; and (c) implementation of any other structured protocol or non-pharmacological intervention specifically targeting BPSD during the study period.

### 2.4. Intervention

The intervention consisted of the implementation of the “Los Filabres” protocol (RPI. 04/2024/578), designed for the management of BPSD. The protocol is based on Virginia Henderson’s model of the 14 basic human needs [19] and the principles of Person-Centered Care (PCC) [20]. The protocol begins with the systematic detection of behavioral changes in residents, guiding caregivers in distinguishing whether these changes correspond to BPSD or to other conditions (e.g., acute confusional states or context-related responses). It then supports a structured assessment and behavioral analysis using the NPI [12] combined with a functional analysis approach. Based on this assessment, potential unmet needs are identified and categorized according to Henderson’s model, allowing for the planning of individualized interventions aimed at addressing these needs. The protocol includes subsequent reassessment to evaluate the effectiveness of the implemented measures, recommending maintenance and prevention strategies when the intervention is successful or returning to the assessment phase if necessary. Its implementation is supported by a clinical decision-making algorithm that facilitates systematic observation, identification of unmet needs, and the development of individualized care strategies, promoting the prevention and reduction of BPSD (Supplementary Figure S1).

### 2.5. Data Collection

Participants were recruited by the multidisciplinary teams at each facility, who were responsible for applying cognitive assessment tools and verifying capacity to provide informed consent. In cases where residents were unable to provide consent due to cognitive impairment, written informed consent was obtained from their legal representatives. Once included, baseline data were collected, including demographic variables (age, sex, and type of dementia) and study variables (number, frequency, severity, clinical relevance, and caregiver-related distress associated with BPSD, as well as psychotropic drug use according to the Anatomical Therapeutic Chemical (ATC) classification system) [21].

The study procedure began with initial contact between the research team and participating facilities, followed by baseline data collection (T0). Staff members at each facility attended a face-to-face training session (4–5 h) delivered by the principal investigator. The protocol was then implemented over a 12-month period, coordinated by a designated professional at each facility (a psychologist). Follow-up assessments were conducted at 6 months (T1) and 12 months (T2) by the same professional responsible for baseline data collection, with continuous monitoring and supervision by the research team.

### 2.6. Measures

#### Neuropsychiatric Inventory (NPI), Spanish Version

The NPI is a widely used instrument for assessing behavioral and psychological symptoms in individuals with dementia and other neurological disorders [12,22]. The Spanish version has demonstrated good internal consistency (Cronbach’s alpha = 0.85) [23]. The NPI includes 12 domains: delusions, hallucinations, agitation/aggression, depression/dysphoria, anxiety, euphoria, apathy/indifference, disinhibition, irritability/lability, aberrant motor behavior, sleep disturbances, and appetite/eating disorders [12,22]. Each domain includes a screening question to determine symptom presence. If present, frequency (F) and severity (S) are rated using 4-point and 3-point Likert scales, respectively. Clinical relevance is calculated as F × S (range: 1–12), with scores ≥ 4 considered clinically significant [22].

### 2.7. Statistical Analysis

Statistical analyses were performed using IBM SPSS Statistics for Windows Version 30.0 (IBM Corp., Armonk, NY, USA). Qualitative variables were described using frequencies and percentages, while quantitative variables were expressed as means and standard deviations (SD). Paired qualitative variables were analyzed using Cochran’s *Q* test. Quantitative variables, following assessment of normality using the Kolmogorov–Smirnov test, were compared using Friedman’s test. Statistical significance was set at *p* < 0.05. No formal correction for multiple comparisons was applied as the analysis focused on pre-planned primary outcomes related to global NPI dimensions and total psychotropic drug counts.

Effect sizes were calculated to assess the magnitude of change between baseline (T0) and the end of follow-up (T2). For quantitative variables, Cohen’s *d* was calculated using the pooled standard deviation of the two assessment points. Effect sizes were interpreted according to Cohen’s criteria: small (0.20), medium (0.50), and large (0.80) [24].

Given its longitudinal observational design, this study acknowledges potential biases such as the Hawthorne effect, where the awareness of being observed may have led to behavioral modifications among both staff and residents during the 12-month period. These methodological considerations, combined with the absence of a control group, necessitate that the findings be interpreted as longitudinal associations rather than direct causal effects.

### 2.8. Ethical Considerations

The study protocol was approved by the Ethics Committee of the Department of Nursing, Physiotherapy, and Medicine of the University of Almería (code EFM 277/23) and conducted in accordance with the principles of the Declaration of Helsinki. Written informed consent was obtained from all participants or, where necessary, from their legal representatives, in accordance with assessments conducted by the facility care teams.

## 3. Results

### 3.1. Participant Characteristics

**Figure 1:**
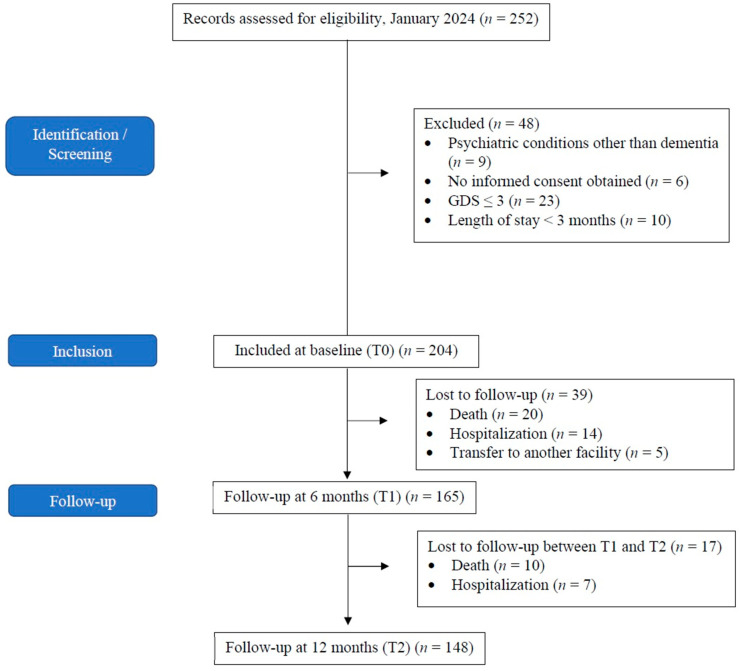
Flowchart of participant selection and follow-up.

A total of 252 residents were initially assessed for eligibility. Of these, 48 were excluded for the following reasons: a GDS score ≤ 3 (*n* = 23), a length of stay of less than three months (*n* = 10), psychiatric conditions other than dementia (*n* = 9), and a lack of informed consent (*n* = 6). Consequently, the baseline sample consisted of 204 participants. At 6 months, the number of evaluated subjects decreased to 165 due to 39 losses during follow-up, broken down as follows: death (*n* = 20), hospitalization (*n* = 14), and transfer to another facility (*n* = 5). Finally, at 12 months, 148 participants completed the study, with 17 additional losses recorded between T1 and T2 due to deaths (*n* = 10) and hospital admissions (*n* = 7) (Figure 1). No adverse events related to the intervention were recorded during the study period. Of the initial sample, 148 participants completed the 12-month follow-up, while 56 did not. To evaluate potential attrition bias, a comparative analysis of baseline characteristics was performed between both groups. No statistically significant differences were found regarding demographic variables, dementia subtypes, baseline cognitive/functional staging, pharmacological treatments, or baseline BPSD metrics (*p* > 0.05), indicating that participant dropout occurred at random.

The sample showed a mean age of 84.49 years (SD = 8.04), and the majority were women (69.1%). Regarding the type of dementia, 42.2% had unspecified cognitive impairment, 33.8% had Alzheimer’s disease, and 9.8% had vascular/mixed dementia. In terms of dementia severity, according to GDS staging, 19.9% of participants were classified as stage 4, 22.1% as stage 5, 37.7% as stage 6, and 21.1% as stage 7, indicating a predominance of moderate-to-severe dementia stages within the study population.

### 3.2. Evolution of BPSD

Table 1 shows the overall evolution of behavioral and psychological symptoms of dementia (BPSD), whereas Table 2 presents the evolution of clinically relevant symptoms (NPI domain score ≥ 4) throughout the study period. At baseline, the overall prevalence of BPSD was almost universal. However, when focusing on clinically relevant symptoms, the proportion of participants was 93.1%, which markedly decreased to 35.8% at 12 months (*p* < 0.001). Participants showed a mean of 4.84 (SD = 2.86) total symptom domains and 3.8 (SD = 2.11) clinically relevant domains. The most prevalent symptoms at baseline were irritability/lability (75.0%), apathy/indifference (59.3%), agitation/aggression (58.3%), and aberrant motor behavior (56.4%), followed by delusions (49.0%) and sleep disturbances (46.6%). A domain-specific analysis showed a significant reduction in the frequency of all evaluated symptoms at 12 months (*p* < 0.05). This decrease was particularly pronounced when considering only clinically relevant symptoms (domain score ≥ 4). In this category, the proportion of participants presenting these symptoms decreased markedly in key domains such as agitation/aggression, apathy/indifference, anxiety, and sleep disturbances (all *p* < 0.001). Other symptoms, including delusions, hallucinations, depression, disinhibition, and irritability, also showed a consistent downward trend from baseline to the end of the study. In contrast, euphoria was the only symptom that did not show a statistically significant reduction despite a decrease in its absolute frequency (*p* > 0.05). Prior to implementation of the “Los Filabres” protocol, 93.1% of participants presented at least one clinically relevant symptom; this proportion was reduced to 35.8% after 12 months. Conversely, the proportion of participants free of clinically relevant symptoms increased significantly from 6.9% at baseline to 64.2% at 12 months (*p* < 0.001). No formal correction for multiple comparisons was applied as the analysis focused on pre-planned primary outcomes related to global NPI dimensions; therefore, the following domain-specific results should be interpreted as exploratory.

Table 3 presents total NPI scores (total BPSD, total frequency, total severity, total clinical relevance, and total caregiver-related distress) across the study period. A significant and progressive reduction was observed across all global NPI dimensions (*p* < 0.001) following implementation of the “Los Filabres” protocol. Effect sizes were large across all dimensions: total frequency (Cohen’s *d* = 1.14), total severity (Cohen’s *d* = 0.97), total caregiver-related distress (Cohen’s *d* = 0.99), and total clinical relevance (Cohen’s *d* = 1.19).

### 3.3. Psychotropic Drug Use

Table 4 shows psychotropic drug use across the three assessment time points (T0, T1, and T2). At baseline, 97.1% of participants were receiving at least one psychotropic drug, with a mean consumption of 2.38 (SD = 1.18) drugs per participant, which decreased to 2.05 (SD = 1.07) when anti-dementia medications were excluded. The most commonly prescribed drug classes were anxiolytics (77.0%) and antipsychotics (72.5%), followed by antidepressants (48.0%), anti-dementia medications (31.4%), and other psychotropic drugs (5.9%). Additionally, 30.9% of participants had “as-needed” (PRN) prescriptions.

Comparative analysis across the three time points showed a significant reduction in overall psychotropic drug use following implementation of the “Los Filabres” protocol (*p* < 0.001). No formal correction for multiple comparisons was applied as the analysis focused on total psychotropic drug counts, and comparisons between drug classes are considered exploratory. Mean drug use decreased from 2.38 (SD = 1.18) at baseline to 1.57 (SD = 0.90) at 12 months, representing a very large effect size (Cohen’s *d* = 0.70; *p* < 0.001). This trend was more pronounced when excluding anti-dementia medications, with a reduction from 2.05 (SD = 1.07) to 1.19 (SD = 0.88) at the end of the study (Cohen’s *d* = 0.87; *p* < 0.001). A marked reduction was observed in anxiolytic use, decreasing from 77.0% to 23.0% (*h* = 1.12; *p* < 0.001), and in antipsychotic use, from 72.5% to 40.5% (Cohen’s *h* = 0.71; *p* < 0.001). In contrast, the use of anti-dementia medications showed a slight but statistically significant increase, from 31.4% to 38.5% (Cohen’s *h* = 0.15; *p* = 0.018), while antidepressants (Cohen’s *h* = 0.01) and other psychotropic drugs (Cohen’s *h* = 0.04) remained stable (*p* > 0.05). The proportion of participants not receiving any psychotropic medication increased significantly from 2.9% to 18.9% (Cohen’s *h* = 0.54; *p* < 0.001). Additionally, “as-needed” prescriptions decreased substantially, from 30.9% at baseline to 1.4% at T2 (Cohen’s *h* = 0.55; *p* < 0.001). Detailed results by individual drugs are presented in Supplementary Table S1.

The results suggest a synchronized reduction in both BPSD clinical relevance and the mean number of psychotropic prescriptions over 12 months.

## 4. Discussion

The primary aim of this study was to evaluate longitudinal changes in behavioral and psychological symptoms of dementia (BPSD) and psychotropic drug use following implementation of the “Los Filabres” protocol in institutionalized individuals with dementia. This structured non-pharmacological intervention integrates the principles of Person-Centered Care with Virginia Henderson’s model of the 14 basic human needs, conceptualizing BPSD as manifestations of unmet needs and guiding interventions toward their identification and resolution. Overall, significant reductions in BPSD and psychotropic drug use were observed during the implementation period, particularly antipsychotics and benzodiazepines. These results are consistent with recent evidence suggesting that BPSD are multifactorial and potentially modifiable phenomena, and that structured non-pharmacological interventions should be considered the first-line approach, especially in long-term care settings [1]. Within this framework, the most effective approaches are those based on a clinical formulation of behavior, integrating personal, physical, emotional, and environmental factors, rather than strategies focused solely on symptom suppression [4,6,25]. Furthermore, previous reviews have emphasized that the effectiveness of these interventions largely depends on staff training and their integration into routine care practice, which are key elements for reducing BPSD and optimizing psychotropic drug use in nursing homes [1,8,25].

Regarding NPI outcomes, the results showed a significant reduction in total scores, as well as in frequency, severity, and caregiver-related distress following implementation of the “Los Filabres” protocol. Improvements were observed both globally and across clinically relevant domains, including agitation/aggression, anxiety, irritability/lability, aberrant motor behavior, depression/dysphoria, sleep disturbances, and psychotic symptoms (delusions and hallucinations). These findings are consistent with previous evidence identifying agitation, irritability, and anxiety as among the domains most responsive to structured non-pharmacological interventions, particularly in institutional settings [1,8,25,26]. In this context, approaches based on behavioral formulation and unmet needs models suggest that personalized interventions integrating physical, emotional, and environmental factors are more effective in reducing BPSD expression than symptom-focused strategies [4,6,8], which is consistent with the results observed in the present study. Evidence from nursing home-based studies supports this interpretation, showing significant reductions in agitation and other BPSD through structured, multicomponent, needs-based interventions, although with considerable variability in effect size and duration [27,28,29]. Similarly, complex programs such as WHELD (Well-Being and Health for People with Dementia) [30], Serial Trial Intervention (STI), STA OP! [28], and behaviorally oriented frameworks such as the DICE approach (Describe, Investigate, Create, Evaluate) [8] have demonstrated significant improvements in NPI domains related to agitation, irritability, and global behavioral symptoms. These models highlight the importance of staff training, systematic case reflection, and longitudinal follow-up. In line with these findings, Thyrian et al. (2017) [31], using a Dementia Care Management approach, reported significant reductions in total NPI scores, supporting the effectiveness of structured models that integrate comprehensive, individualized care planning, and coordinated care in residential settings. One of the most relevant findings of this study is the magnitude of the observed changes. The effect sizes confirm a large clinical impact (Cohen’s *d* ranging from 0.96 to 1.19). While these values are more conservative than initially calculated, they still indicate a robust and highly significant improvement compared to many non-pharmacological interventions reported in previous literature, which typically show small to moderate effects [30,32,33]. Several factors may explain this difference, including the structured nature of the intervention, its sustained implementation over time, the integration of staff training, and its grounding in a needs-based conceptual framework. Together, these elements may help explain the magnitude of the observed changes, contributing to a more global and sustained impact on symptom expression. The observed reductions in symptom frequency, severity, and overall burden suggest that the protocol may operate at a global level in BPSD expression, rather than targeting isolated symptoms. The reduction in the number of participants with clinically relevant symptoms further supports the hypothesis that the intervention may contribute to improved behavioral stability and reduced symptom-related distress.

Regarding pharmacological outcomes, a large effect size was observed in the reduction of anxiolytic and antipsychotic use, suggesting that the protocol may be associated with reduced reliance on chemical restraint in favor of behavioral management strategies. In contrast, no relevant changes were observed in anti-dementia medications or other psychotropic drugs not directly related to behavioral symptom management, indicating that the intervention did not substantially affect these treatments. These findings are consistent with current recommendations, which advocate non-pharmacological interventions as first-line strategies for managing BPSD, reserving psychotropic drugs for severe or refractory cases and for limited durations with regular reassessment [1,2,8,25]. Evidence consistently shows that psychotropic drugs have modest efficacy in BPSD, while their safety profile is concerning, particularly for antipsychotics and benzodiazepines, which are associated with increased risks of sedation, falls, cerebrovascular events, and mortality [2,8]. This reinforces the need for cautious prescribing and deprescribing strategies when clinically appropriate. Evidence from nursing home studies supports that reductions in BPSD through structured non-pharmacological interventions can be achieved without increasing psychotropic drug use. For example, Gillis et al. (2023) [34] reported significant improvements in BPSD using a needs-based care approach, without significant changes in psychotropic prescriptions, suggesting that behavioral improvement was not driven by medication. Other structured programs have demonstrated a more direct impact on prescribing patterns. The STA OP! model was associated with reductions in antidepressant use and stabilization of other psychotropic prescriptions while maintaining behavioral improvements [28], whereas the intervention described by Zwijsen et al. (2014) [29] prioritized the identification of underlying causes before pharmacological intervention. Additionally, Thyrian et al. (2017) [31] showed that structured interventions may lead not necessarily to indiscriminate medication reduction, but rather to optimization of pharmacotherapy, improving BPSD without increasing potentially inappropriate medications. Taken together, these findings support the interpretation that the reduction in psychotropic drug use observed in this study reflects a process of therapeutic rationalization rather than arbitrary withdrawal, reinforcing the role of structured non-pharmacological interventions as a central strategy in dementia care. These findings suggest that implementing such protocols at a policy level could reduce reliance on chemical restraints and lower healthcare costs associated with psychotropic-related adverse events in nursing homes. A primary original contribution of this study is that the “Los Filabres” protocol is the first to integrate Virginia Henderson’s 14 basic human needs model into a structured clinical decision-making algorithm for BPSD. Our protocol offers a unique operational guide by grounding behavioral analysis in a widely recognized nursing framework. This integration allows for a more precise identification of unmet needs and provides a systematic, evidence-based pathway specifically adapted to the daily clinical complexity of residential settings.

From a clinical perspective, these findings support the relevance of addressing BPSD from an unmet needs perspective, representing a shift from symptom control toward understanding the meaning of behavior. The “Los Filabres” protocol operationalizes this approach through a structured, accessible, and evidence-based intervention that facilitates consistent and clinically informed decision-making. The inclusion of a decision-making algorithm represents a key strength, enabling both rapid application in routine care and more reflective clinical analysis, thereby promoting consistency and continuity of care in residential settings. Strengths of this study include the combined evaluation of behavioral and pharmacological outcomes, as well as its implementation in real-world long-term care settings, enhancing the external validity and applicability of the findings. Despite these findings, several limitations must be considered when interpreting the results. The absence of a randomized control group constitutes the main limitation of this study. However, this design choice was strictly dictated by ethical and logistical constraints inherent to long-term care settings for individuals with moderate-to-severe dementia. From an ethical perspective, withholding a potentially beneficial, person-centered non-pharmacological intervention from a subset of vulnerable residents was considered unfeasible. Furthermore, from a methodological standpoint, allocating residents within the same facility to a control group would have introduced a high risk of contamination bias, as the multidisciplinary staff was collectively trained to implement the “Los Filabres” protocol. To mitigate this absence, a rigorous longitudinal design was adopted, allowing participants to serve as their own comparison over time. Due to its longitudinal observational design, it is not possible to establish a direct causal relationship between the implementation of the “Los Filabres” protocol and the reduction of symptoms or medication use. Consequently, the results should be interpreted as positive longitudinal associations rather than definitive causal effects. A limitation of this study is the diagnostic heterogeneity of the sample, which included various types of dementia and unspecified cognitive impairment. Although the “Los Filabres” protocol utilizes a transdiagnostic approach based on universal needs, the response to non-pharmacological interventions could vary depending on the dementia subtype, thereby limiting the specificity of the findings. Furthermore, a significant methodological limitation is the high risk of detection bias, as no independent masking (blinding) was employed during the assessment of outcomes. The facility psychologists and multidisciplinary teams served a dual role as both the implementers of the protocol and the evaluators of the NPIs and medication records. Therefore, these findings may be influenced by uncontrolled factors such as the natural progression of dementia, internal organizational changes in the nursing homes, or observer expectation bias. Additionally, as stated in Section 2, the Hawthorne effect is considered an inherent factor in this type of observational design. Furthermore, while the intervention included structured staff training and a clinical decision-making algorithm, the absence of quantitative fidelity measures to monitor protocol adherence across the five facilities is a notable limitation. Consequently, although the data indicate a viable and valuable clinical trend, future research must implement blinded independent evaluators and randomized controlled trials to minimize these biases, incorporate formal implementation tools, and isolate the specific influence of the intervention from other contextual variables.

## 5. Conclusions

The implementation of the “Los Filabres” protocol was associated with a reduction in BPSD and psychotropic drug use during the 12-month follow-up period in institutionalized individuals with dementia. Structured, person-centered non-pharmacological interventions based on the identification of unmet needs could contribute to improving the management of these symptoms and promoting safer and more individualized care in nursing home settings.

## Figures and Tables

**Table 1:** Longitudinal changes in overall BPSD prevalence (NPI).

BPSD (Overall Prevalence)				
BPSD Domains (NPI)	Baseline (T0) (*n* = 204)	6 Months (T1) (*n* = 165)	12 Months (T2) (*n* = 148)	*p*-Value
Delusions	100 (49.0%)	57 (34.5%)	45 (30.4%)	*p* < 0.001 ^(a)^
Hallucinations	25 (12.3%)	20 (12.1%)	8 (5.4%)	*p* = 0.001 ^(a)^
Agitation/Aggression	119 (58.3%)	64 (40.6%)	33 (22.3%)	*p* < 0.001 ^(a)^
Depression/Dysphoria	85 (41.7%)	55 (33.3%)	29 (19.6%)	*p* < 0.001 ^(a)^
Anxiety	68 (33.3%)	43 (26.1%)	15 (10.1%)	*p* < 0.001 ^(a)^
Euphoria	7 (3.4%)	5 (3.0%)	0 (0.0%)	*p* < 0.05 ^(a)^
Apathy/Indifference	121 (59.3%)	85 (51.7%)	75 (50.7%)	*p* < 0.05 ^(a)^
Disinhibition	45 (22.1%)	30 (18.2%)	9 (6.1%)	*p* < 0.001 ^(a)^
Irritability/Lability	153 (75.0%)	88 (53.3%)	54 (36.5%)	*p* < 0.001 ^(a)^
Aberrant Motor Behavior	115 (56.4%)	67 (40.6%)	33 (22.3%)	*p* < 0.001 ^(a)^
Sleep Disturbances	95 (46.6%)	38 (23.0%)	18 (12.2%)	*p* < 0.001 ^(a)^
Appetite/Eating Disorders	63 (30.9%)	30 (18.2%)	6 (4.1%)	*p* < 0.001 ^(a)^
Total BPSD, mean (SD)	4.84 (2.86)	3.28 (2.15)	2.10 (2.81)	*p* < 0.001 ^(b)^

Data are presented as *n* (%) or mean (SD), SD: Standard deviation, ^(a)^ Cochran’s *Q* test, ^(b)^ Friedman test.

**Table 2:** Longitudinal changes in clinically relevant BPSD (NPI domain score ≥ 4).

Clinically Relevant BPSD (NPI Domain Score [Frequency × Severity] ≥ 4)				
BPSD Domains (NPI)	Baseline (T0) (*n* = 204)	6 Months (T1) (*n* = 165)	12 Months (T2) (*n* = 148)	*p*-Value
Delusions	75 (36.8%)	30 (18.2%)	17 (11.5%)	*p* < 0.001 ^(a)^
Hallucinations	23 (11.3%)	13 (7.9%)	3 (2.0%)	*p* < 0.001 ^(a)^
Agitation/Aggression	97 (47.5%)	27 (16.4%)	9 (6.1%)	*p* < 0.001 ^(a)^
Depression/Dysphoria	60 (29.4%)	26 (15.8%)	7 (4.7%)	*p* < 0.001 ^(a)^
Anxiety	53 (26.0%)	20 (12.1%)	4 (2.7%)	*p* < 0.001 ^(a)^
Euphoria	2 (1.0%)	1 (0.6%)	0 (0.0%)	*p* > 0.05 ^(a)^
Apathy/Indifference	110 (53.9%)	53 (32.1%)	25 (16.9%)	*p* < 0.001 ^(a)^
Disinhibition	33 (16.2%)	13 (7.9%)	3 (2.0%)	*p* < 0.001 ^(a)^
Irritability/Lability	125 (61.3%)	49 (29.7%)	15 (10.1%)	*p* < 0.001 ^(a)^
Aberrant Motor Behavior	97 (47.5%)	26 (15.8%)	5 (3.4%)	*p* < 0.001 ^(a)^
Sleep Disturbances	71 (34.8%)	17 (10.3%)	4 (2.7%)	*p* < 0.001 ^(a)^
Appetite/Eating Disorders	56 (27.5%)	18 (10.9%)	2 (1.4%)	*p* < 0.001 ^(a)^
Total clinically relevant BPSD, mean (SD)	3.93 (2.11)	1.78 (1.05)	0.64 (0.30)	*p* < 0.001 ^(b)^
Participants with ≥1 clinically relevant symptom	190 (93.1%)	119 (72.1%)	53 (35.8%)	*p* < 0.001 ^(a)^
No clinically relevant symptoms	14 (6.9%)	46 (27.9%)	95 (64.2%)	*p* < 0.001 ^(a)^

Data are presented as *n* (%) or mean (SD), Clinically relevant BPSD defined as NPI domain score (frequency × severity) ≥ 4, SD: Standard deviation, ^(a)^ Cochran’s *Q* test, ^(b)^ Friedman test.

**Table 3:** Longitudinal changes in NPI scores and standardized effect size estimates during the implementation period of the “Los Filabres” protocol.

BPSD (NPI)	Baseline (T0) (*n* = 204)	6 Months (T1) (*n* = 165)	12 Months (T2) (*n* = 148)	Effect Size (Cohen’s *d*, T0–T2) ^†^	*p*-Value ^(a)^
Total BPSD score	4.84 (2.86)	3.28 (2.15)	2.10 (2.81)	0.96	*p* < 0.001
Total frequency	13.63 (9.56)	7.59 (4.46)	3.70 (2.30)	1.14	*p* < 0.001
Total severity	10.34 (8.40)	5.48 (3.33)	3.05 (2.21)	0.97	*p* < 0.001
Total clinical relevance	30.64 (22.53)	13.23 (10.01)	5.65 (3.90)	1.19	*p* < 0.001
Total caregiver-related distress	15.32 (12.65)	7.58 (5.48)	3.77 (2.30)	0.99	*p* < 0.001

Data are presented as mean (SD), SD: Standard deviation, ^†^ Cohen’s *d* estimates reflect the magnitude of change calculated using the pooled standard deviation, ^(a)^ Friedman test.

**Table 4:** Changes in psychotropic drug use by therapeutic group during the implementation period of the “Los Filabres” protocol.

Psychotropic Drugs	Baseline (T0) (*n* = 204)	6 Months (T1) (*n* = 165)	12 Months (T2) (*n* = 148)	Effect Size (Cohen’s h/d, T0–T2)	*p*-Value
Participants receiving at least one psychotropic drug	198 (97.1%)	145 (87.9%)	120 (81.1%)	0.55 ^†^	*p* < 0.001 ^(a)^
Mean number of psychotropic drugs	2.38 (1.18)	2.03 (1.10)	1.57 (0.90)	0.70 ^††^	*p* < 0.001 ^(b)^
Mean number of psychotropic drugs (excluding anti-dementia medications)	2.05 (1.07)	1.64 (0.98)	1.19 (0.88)	0.87 ^††^	*p* < 0.001 ^(b)^
Participants not receiving psychotropic drugs	6 (2.9%)	20 (12.1%)	28 (18.9%)	0.54 ^†^	*p* < 0.001 ^(a)^
Anxiolytics	157 (77.0%)	92 (55.8%)	34 (23.0%)	1.12 ^†^	*p* < 0.001 ^(a)^
Antipsychotics	148 (72.5%)	91 (55.2%)	60 (40.5%)	0.71 ^†^	*p* < 0.001 ^(a)^
Antidepressants	98 (48.0%)	79 (47.9%)	70 (47.3%)	0.01 ^†^	*p* > 0.05 ^(a)^
Anti-dementia medications	64 (31.4%)	61 (37.0%)	57 (38.5%)	0.15 ^†^	*p* = 0.018 ^(a)^
Other psychotropic drugs	12 (5.9%)	13 (7.9%)	12 (8.1%)	0.04 ^†^	*p* > 0.05 ^(a)^
As-needed (PRN) prescriptions	63 (30.9%)	26 (15.8%)	2 (1.4%)	0.55 ^†^	*p* < 0.001 ^(a)^

Data are presented as mean (SD) or n (%), SD: Standard deviation, ^†^ Cohen’s *h*, ^††^ Cohen’s *d* estimates reflect the magnitude of change calculated using the pooled standard deviation, ^(a)^ Cochran’s *Q* test, ^(b)^ Friedman test.

## Data Availability

The data presented in this study are available from the corresponding author upon reasonable request.

## Supplementary Materials

The following supporting information can be downloaded at: https://www.mdpi.com/article/10.3390/healthcare14131934/s1, Figure S1: BPSD Decision-Making Algorithm; Table S1: Changes in psychotropic drug use by individual active substances following implementation of the “Los Filabres” protocol.

## Abbreviations

The following abbreviations are used in this manuscript:

BPSD	Behavioral and psychological symptoms of dementia
PCC	Person-Centered Care
NPI	Neuropsychiatric Inventory
GDS	Global Deterioration Scale
RPI	Intellectual Property Registry
ATC	Anatomical Therapeutic Chemical
PRN	pro re nata (as needed)
SD	Standard deviation
T0	Baseline
T1	6 months
T2	12 months
F	Frequency
S	Severity
