# Supplementary material for: Evaluation of the “Los Filabres” Protocol on Behavioral and Psychological Symptoms of Dementia and Psychotropic Drug Use in Nursing Home Residents
Source: Healthcare (Basel). 2026 Jul 1;14(13):1934. doi: 10.3390/healthcare14131934 (PMC13361222; doi:10.3390/healthcare14131934)
Supplement: Supplementary file 1 [file healthcare-14-01934-s001.zip › healthcare-4273316-supplementary.pdf]

BPSD Decision-Making Algorithm

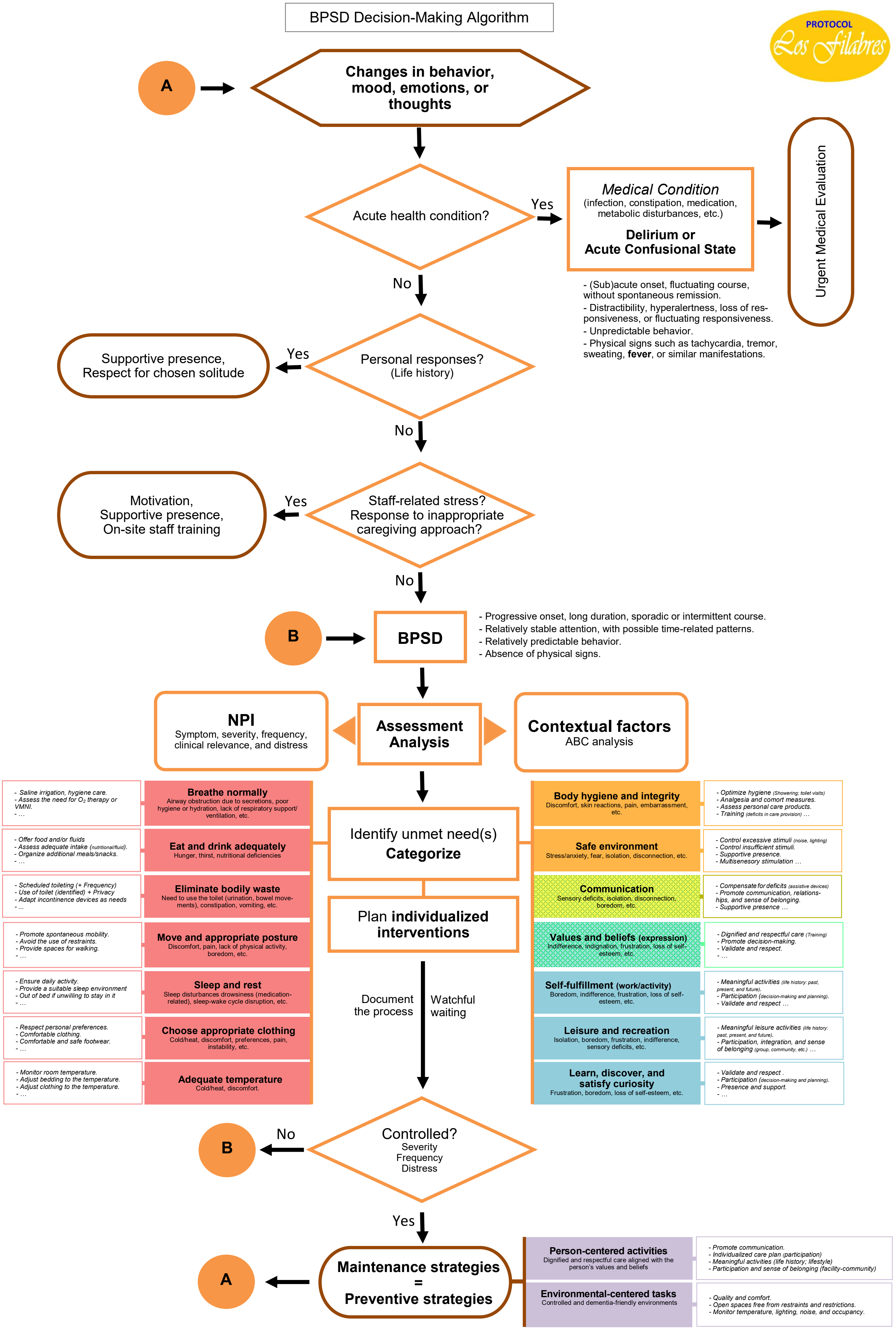

Supplementary Table S1: *Changes in psychotropic drug use by individual active substances following implementation of the "Los Filabres" protocol.*

Psychotropic drugs	Baseline (T0)	6 months (T1)	12 months (T2)	p-value
**Antidepressants**				
**Citalopram**	15 (7.4)	9 (5.5)	8 (5.4)	$p < 0.05$ [a]
**Escitalopram**	4 (2.0)	3 (1.8)	3 (2.0)	$p = 0.37$ [a]
**Sertraline**	20 (9.8)	23 (13.9)	23 (15.5)	$p < 0.05$ [a]
**Mirtazapine**	8 (3.9)	6 (3.6)	5 (3.4)	$p = 0.37$ [a]
**Trazodone**	46 (22.5)	41 (24.8)	35 (23.6)	$p = 0.59$ [a]
**Venlafaxine**	2 (1.0)	1 (0.6)	1 (0.7)	$p = 1.0$ [a]
**Duloxetine**	1 (0.5)	1 (0.6)	1 (0.7)	$p = 1.0$ [a]
**Amitriptyline**	6 (2.9)	1 (0.6)	1 (0.7)	$p < 0.05$ [a]
**Vortioxetine**	8 (3.9)	3 (1.8)	2 (1.4)	$p < 0.05$ [a]
**Other antidepressants**	5 (2.5)	1 (1.8)	2 (1.4)	$p = 0.37$ [a]
**Anxiolytics**				
**Lorazepam**	87 (42.6)	32 (19.4)	13 (8.8)	$p < 0.001$ [a]
**Lormetazepam**	42 (20.6)	29 (17.6)	11 (7.4)	$p < 0.001$ [a]
**Alprazolam**	37 (18.1)	17 (10.3)	2 (1.4)	$p < 0.001$ [a]
**Bromazepam**	17 (8.3)	3 (1.8)	2 (1.4)	$p < 0.001$ [a]
**Diazepam**	11 (5.4)	1 (0.6)	0 (0.0)	$p < 0.001$ [a]
**Midazolam**	4 (2.0)	2 (1.2)	0 (0.0)	$p = 0.05$ [a]

**Clonazepam**	14 (6.9)	9 (5.5)	5 (3.4)	$p < 0.05$ [(a)]
**Other anxiolytics**	11 (5.4)	6 (3.6)	1 (0.7)	$p < 0.05$ [(a)]
**Antipsychotics**				
**Haloperidol**	24 (11.8)	4 (2.4)	0 (0.0)	$p < 0.001$ [(a)]
**Risperidone**	12 (5.9)	6 (3.6)	6 (4.1)	$p = 0.64$ [(a)]
**Quetiapine**	124 (60.8)	75 (45.5)	50 (33.8)	$p < 0.001$ [(a)]
**Olanzapine**	7 (3.4)	2 (1.2)	0 (0.0)	$p < 0.05$ [(a)]
**Levomepromazine**	5 (2.5)	4 (2.4)	0 (0.0)	$p < 0.05$ [(a)]
**Other antipsychotics**	9 (4.4)	6 (3.6)	0 (0.0)	$p < 0.05$ [(a)]
**Anti-dementia medications**				
**Memantine**	28 (13.7)	29 (17.6)	27 (18.2)	$p = 0.08$ [(a)]
**Donepezil**	30 (14.7)	30 (18.2)	29 (19.6)	$p = 0.72$ [(a)]
**Rivastigmine**	19 (9.6)	10 (6.1)	8 (5.4)	$p = 0.14$ [(a)]
**Galantamine**	1 (0.5)	1 (0.6)	1 (0.7)	$p = 1.0$ [(a)]
**Other psychotropic drugs**				
**Levodopa**	9 (4.4)	8 (4.8)	8 (5.4)	$p = 1.0$ [(a)]
**Carbidopa**	8 (3.9)	6 (3.6)	6 (4.1)	$p = 0.37$ [(a)]
**Eslicarbazepine**	2 (1.0)	2 (1.2)	2 (1.4)	$p = 1.0$ [(a)]
**Clomethiazole**	2 (1.0)	0 (0.0)	0 (0.0)	$p = 0.14$ [(a)]

Data are presented as n (%)
[(a)] Cochran’s Q test
